# Intragastric driveline transposition of a left ventricular assist device: a case report

**DOI:** 10.1093/jscr/rjag217

**Published:** 2026-04-04

**Authors:** Laura Stöffler, Nikolaos Cholevas, Evgenij Potapov, Wenzel Schöning

**Affiliations:** Department of Surgery, Charité—Universitätsmedizin Berlin, cooperate member of Freie Universität Berlin and Humboldt-Universität zu Berlin, Augustenburger Platz 1, 13353 Berlin, Germany; Department of Cardiothoracic and Vascular Surgery, Deutsches Herzzentrum der Charité (DHZC), Augustenburger Platz 1, 13353 Berlin, Germany; DZHK (German Centre for Cardiovascular Research), Partner Site Berlin, Augustenburger Platz 1, 13353 Berlin, Germany; Department of Cardiothoracic and Vascular Surgery, Deutsches Herzzentrum der Charité (DHZC), Augustenburger Platz 1, 13353 Berlin, Germany; DZHK (German Centre for Cardiovascular Research), Partner Site Berlin, Augustenburger Platz 1, 13353 Berlin, Germany; Department of Surgery, Charité—Universitätsmedizin Berlin, cooperate member of Freie Universität Berlin and Humboldt-Universität zu Berlin, Augustenburger Platz 1, 13353 Berlin, Germany

**Keywords:** LVAD, gastric perforation, interdisciplinary surgery

## Abstract

Implantation of left ventricular assist devices (LVADs) is increasingly utilized, prolonging survival of patients with advanced heart failure. We herein report a severe complication of gastric perforation with an intragastric transposition of an LVAD driveline (DL). The patient was admitted to interdisciplinary surgery, where the DL was successfully removed from the stomach and the gastric perforation addressed by performing an atypical gastric resection. This is the first reported case of an intragastric transposition of DL and LVAD pump of a HeartMate II LVAD. This case underscores the importance of interdisciplinary efforts in the treatment of LVAD associated complications as well as regular gastrointestinal monitoring in LVAD patients.

## Introduction

Left ventricular assist devices (LVADs) are utilized in patients with advanced heart failure [1]. The most common LVAD associated complications are driveline (DL) infections as well as bleeding and thromboembolic complications. Bleeding occurs particularly in the gastrointestinal tract, often due to arteriovenous malformation [2, 3]. We present the first case of gastric decubitus with gastric perforation and intragastric transposition of the DL of one of the LVADs currently in use.

## Case report

A 63-year-old male patient, with history of 13-year support on an axial continuous flow LVAD HeartMate II (Abbott, IL, USA) was admitted for further evaluation and treatment of a *Streptococcus anginosus* bloodstream infection of unknown origin. He had previously completed a 3-week course of empiric antibiotic treatment with ampicillin/sulbactam for an infection presenting with fever, shivering, and elevated infectious parameters. Two weeks ago, the patient reported a single episode of non-hemorrhagic vomiting. Further abdominal symptoms were denied and the exit-side of the DL presented irritation-free.

The LVAD was implanted due to ischemic cardiomyopathy. Co-morbidities included peripheral artery disease and diabetes mellitus type 2 with diabetic nephropathy. After the HeartMateII implantation, multiple episodes of gastrointestinal bleeding (2012/2017/2021) and a chronic DL-infection with *Staphylococcus anginosus* and *Pseudomonas aeruginosa* with abscess formation were reported. Due to the latter, he received a DL-transposition in 2022, where an intraoperatively produced small defect in the silicone protective sheath of the intracorporeal part of the DL was immediately intraoperatively encased with plastic covers [4].

**Figure 1:**
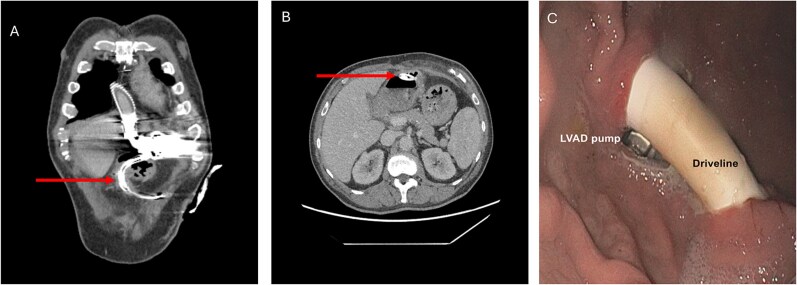
Preoperative CT scan (A, B) and esophagogastroduodenoscopy (C) revealing intragastric DL transposition. The arrow points at the DL.

The obtained blood cultured revealed the presence of *S. anginosus*. As infectious parameters were persistently elevated after empirical antibiotic treatment escalation to piperacillin/tazobactam with no specific clinical focus, a computed tomography (CT) scan was performed revealing free intra-abdominal air and an intragastric transposition of the LVAD-DL (Fig. 1A and B). This was confirmed by esophagogastroduodenoscopy, which revealed a short-range intragastric course of the DL with its entry and exit sides, as well as visible components of the pump (Fig. 1B and C). Next to the intragastric DL, a gastric ulcer measuring ~10 mm was identified.

## Treatment

**Figure 2:**
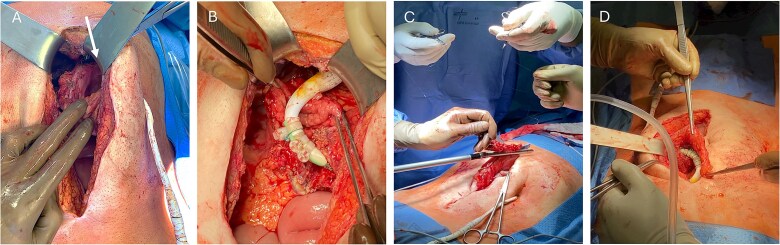
Stomach after preparation with intragastric DL course, the pump is indicated with a white arrow (A). Careful gastric resection and relocation of the DL from the stomach (B) followed by stapling (C) and envelopment with greater omentum (D).

The patient was immediately scheduled for interdisciplinary surgery. After upper median re-laparotomy and extensive adhesiolysis, the gastric insertion and exit sites of the migrated DL were circumferentially dissected from the surrounding tissue (Fig. 2A). An atypical gastric resection measuring ~8 cm was performed that removed the involved gastric segments (Fig. 2B). Resection was performed using the ECHELON FLEX (Ethicon) stapling device and reinforced with a single line manual suture (Fig. 2C). This was followed by extensive intraabdominal lavage. The DL was then dissected from the abdominal wall at the peritoneal site, disconnected, and externalized on the contralateral side before reconnection to the pump controller. Plastic covers (Fig. 2B) previously placed (during the DL transposition in 2022) were removed and the DL was enveloped with greater omentum (Fig. 2D).

The anti-infective treatment was continued with piperacillin/tazobactam and augmented with Caspofungin. After surgery, the patient developed superficial wound infection which was addressed with an epicutaneous vacuum-assisted closure. Microbiological samples of the wound and DL detected a significant growth of *Candida glabrata*, *Candida albicans,* and *P. aeruginosa*, leading to directed anti-infective therapy with rifampicin, voriconazole, and doxycycline. Infectious parameters subsequently decreased and the patient was discharged home in overall good condition 21 days after surgery.

Six months later, the patient was readmitted with a further episode of DL infection with a cable fracture prompting emergency pump exchange. He died due to a septic shock following the LVAD pump exchange to HeartMate 3 employing a cardio-pulmonary bypass.

The patient provided written informed consent for publications.

## Discussion and conclusion

Long-term survival in LVAD patients is improving, representing an essential treatment option for patients with end-stage heart failure not eligible for heart transplantation [5]. However, LVAD associated complications remain frequent and require complex treatment approaches including surgery, demanding advanced interdisciplinary efforts [6]. In our center, ~3% of the LVAD patients required DL-transposition due to severe infection (70 transpositions in total).

Until now, LVAD associated gastric perforation has only been reported in two cases for large bulky intraabdominally implanted ThermoCardiosystems (TCI) LVAD (Thoratec, Plesanton, USA) and HeartMate I [7, 8]. In the first instance extrinsic compression by the LVAD caused gastric ulcer formation and perforation, whereas in the second case the LVAD ran through the anterior wall of the stomach covered by omentum. A case of gastric compression caused by the HeartMateII LVAD without perforation was described in 2016 [9].

In the present case, the patient survived surgery and initially left the hospital in good overall condition. It remains unclear whether the perforation was primarily caused by (i) gastric ulceration, (ii) mechanical erosion from compression of the chronically infected DL and plastic repair covers, or both. This rare yet severe gastrointestinal complication underlines the importance of recognizing potential risk factors such as chronic DL infection, malnutrition, and pre-existing gastric pathology, all of which may increase the risk of pressure necrosis and gastric ulceration. Also, measures to prevent DL infection are essential including an accurate positioning of the DL, its secure fixation to minimize traction and pressure as well as standardized exit-site care [10].

As the present report indicates, diagnosis of gastric perforation in LVAD patients can be very challenging, as the absence of abdominal symptoms does not preclude this potentially fatal diagnosis. In case of sustained elevated infectious parameters, a lower threshold for cross-sectional imaging should be considered in this patient population.

We reported here a first case of HeartMateII associated gastric perforation with successful initial treatment. This case underscores that the management of DL infections and gastrointestinal complications in general, and especially in such complicated cases, should be performed by an interdisciplinary team of highly specialized and experienced cardiac and visceral surgeons.
